# Luminescence behavior of silicon and carbon nanoparticles dispersed in low-polar liquids

**DOI:** 10.1186/1556-276X-7-365

**Published:** 2012-07-02

**Authors:** Yury V Ryabchikov, Sergei A Alekseev, Vladimir V Lysenko, Georges Bremond, Jean-Marie Bluet

**Affiliations:** 1 Institut des Nanotechnologies de Lyon (INL), UMR-5270, CNRS, Université de Lyon, INSA de Lyon, bat. Blaise Pascal, 7 av. Jean Capelle, Bat. Blaise Pascal, Villeurbanne, F-69621, France; 2 PN Lebedev Physical Institute of Russian Academy of Sciences, 53 Leninskii Prospekt, Moscow, 199991, Russia; 3 Faculty of Chemistry, National Taras Shevchenko University, 64 Volodymyrska Str, Kyiv, 01601, Ukraine

**Keywords:** Silicon nanoparticles, Carbon nanoparticles, Photoluminescence, Chemical modification, Alkyl groups, Low-polar liquids, Temperature dependence

## Abstract

A comparative photoluminescence analysis of as-prepared and chemically modified (by alkyl chains -C_18_H_37_) silicon and carbon nanoparticles dispersed in low-polar liquids is reported. Influence of the low-polar liquid nature and ambient temperature on photoluminescence of the nanoparticles has been investigated from the point of view of their possible application as thermal nanoprobes.

## Background

Low-dimensional structures present considerable interest for researchers because of their new and unique optical and electronic properties in comparison with bulk materials. For example, various nanoparticles (NPs) are involved in different applications such as electronics [1], photovoltaic [2], biology [3], liquid crystals [4] etc. Photoluminescence (PL) is one of the important phenomena taking place in semiconductor NPs. It is very sensitive to various experimental conditions, such as molecular ambient environment, temperature, pressure, and so on. In particular, thermally induced PL effects are of special interest for temperature probing at nanoscale in various media. For example, different materials are reported to be used as thermal-sensitive nanoprobes [5] and nanothermometers [6].

A comparative analysis of the influence of high- and low-polar liquids (LPLs) on PL of Si NPs has already been reported [7]. The low-polar liquids were reported to be used instead of the high-polar liquids in order to prevent PL quenching of the NPs. However, stable colloidal solutions of the as-prepared Si NPs could not be obtained in LPLs because of their oleophobic surface. Thus, surface chemical modification of the NPs by different alkyl groups can be the simplest way to overcome this problem [8].

In this work, we have studied the influence of LPLs, ambient temperature, and continuous laser irradiation on PL behavior of Si and C NPs in view of their temperature-sensing applications.

## Methods

Chemical anodic etching of *p*-type 10-Ω·cm (100)-oriented Si wafer has been used for the preparation of nano-Si powder. Silicon substrate was etched in a solution containing 1:1 volume mixture of HF (48%) and anhydrous ethanol. Anodic current density was 45 mA/cm^2^, and etching time is 50 min. A permanent stirring of the etching solution was applied in order to evacuate hydrogen bubbles formed during the etching process. The anodization was performed in a Teflon cell with a copper electrode as a backside contact. The counter electrode was made of platinum. After the etching, a highly porous network constituted by numerous interconnected nanocrystals was formed. The sample was washed with anhydrous ethanol several times, dried in ambient air, and then scratched out from the wafer. Mechanical dry grinding of the formed free nanoporous layer transformed it into a powder state.

Thermal hydrosilylation approach, described in details in the review [9], was used for the grafting of alkyl groups (−C_18_H_37_) onto the surface of Si nanoparticles. Freshly prepared powder (30 mg) was treated with 1.5 mL of 1-octadecene at 150°C for 16 h under magnetic stirring in N_2_ atmosphere. The reaction mixture was cooled to room temperature and centrifuged for 10 min at 20,000 × *g* to remove large particles.

Photoluminescent C NPs with the surface substantially covered with carboxylic acid groups were prepared by means of electrochemical dissolution of SiC wafers. To functionalize their surface with -C_18_H_37_ groups, the powder of C NPs was refluxed for 1 h with the excess of octadecylamine C_18_H_37_NH_2_ in anhydrous *o*-xylene; according to FTIR data, the amide bonds are formed between C NPs and C_18_H_37_NH_2_. The liquid was then slowly distilled out (in the stream of N_2_, bath temperature did not exceed 155°C); afterwards, a small portion of *o*-xylene was added and distilled out to remove residues of H_2_O which form the reaction mixture. To workup alkylated carbon nanoparticles, residue after solvent evaporation was redispersed in *n*-hexane and extracted several times by 0.5 mol/L HCl in methanol to remove excess of C_18_H_37_NH_2_, then washed twice with H_2_O, centrifuged (20,000 × *g*, 2 × 10 min) to remove any products, insoluble in hexane, than the hexane solution allowed to evaporate and dried in air at 85°C. Brown waxy residue of alkylated C NPs is readily dispersible in non-polar solvents.

Photoluminescence excitation of all NPs was performed by means of argon-ion laser with excitation photon energy at 2.54 eV. The laser spot area was about 10^−4^ cm^−2^. Additionally, laser diode with the excitation photon energy at 3.62 eV has been used for the excitation of C-C_18_H_37_ NPs. All colloidal solutions of the NPs were put in a UV transparent quartz cuvette in a volume of 1 mL. Temperature dependence PL measurements were carried out using ViscoTerm VT100 temperature cell (VISCO Pump & Seal Group, Brooklyn, Victoria, Australia). In this case, the colloidal solutions were placed in a special sample holder. HORIBA Jobin Yvon (Edison, NJ, USA) iHR-320 monochromator was used for spectral decomposition of the PL signal. The PL spectra of the colloidal solutions were detected by nitrogen-cooled CCD camera HORIBA Symphony 1024 × 256 (HORIBA Jobin Yvon Edison, NJ, USA). All PL spectra were corrected using the spectral response function of the whole experimental setup.

## Results and discussion

**Figure 1:**
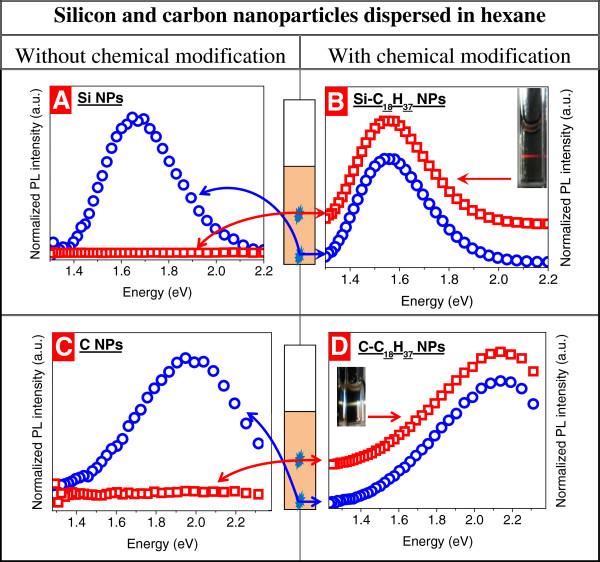
**Room-temperature PL spectra of NPs in hexane.** Si (**A**), Si-C_18_H_37_ (**B**), C (**C**), C-C_18_H_37_ (**D**) NPs in hexane collected for an excitation of the solution volume in the bottom and in the center. The spectra in (**B**) and (**D**) were vertically stacked for clearness. The insets in (**B**) and (**D**) show, respectively, red PL of Si-C_18_H_37_ and orange PL of C-C_18_H_37_ NPs. Excitation energy is 2.54 eV.

Figure 1 illustrates the influence of surface chemical modification by alkyl groups of silicon (Figure 1A,B) and carbon (Figure 1C,D) NPs on their PL properties and quality of their dispersion in hexane. For the as-prepared NPs, no PL spectra are observed if the excitation is applied in the center of solution volume (Figure 1A,C), while typical PL spectra of Si and C NPs were detected for the case of the excitation of the solution bottom (Figure 1B,D). Quite broad (360 meV) full width at half maximum of these spectra corresponds to relatively large size distribution of the studied NPs. The observed very poor dispersion of the as-prepared Si and C NPs in hexane can be explained by their oleophobic surface chemistry leading to their accumulation at the solution bottom. In contrast, oleophilic surface chemistry of the NPs achieved by means of alkyl groups allows their efficient in-volume dispersion in LPLs. Indeed, as one can see, the PL spectra of the colloidal solutions based on both Si-C_18_H_37_ and C-C_18_H_37_ NPs are independent on the excitation position of the solution volume (Figure 1B,D) in which they are homogeneously dispersed.

It is worth to note that the performed surface chemical modification by the same alkyl groups impacts differently surface states of the Si and C NPs and, consequently, their original PL spectra. As one can state, the red (110 meV) and blue (160 meV) shifts of the PL maxima occur for Si and C NPs, respectively. This fact can be explained by different influences of the grafted -C_18_H_37_ groups on density of the band tail electronic states (surface states) involved in radiative transitions of the charge carriers photogenerated in the studied NPs. In particular, the surface chemical modification by alkyl groups leads to increase of this state density for Si NPs and to its decrease for C NPs.

**Figure 2:**
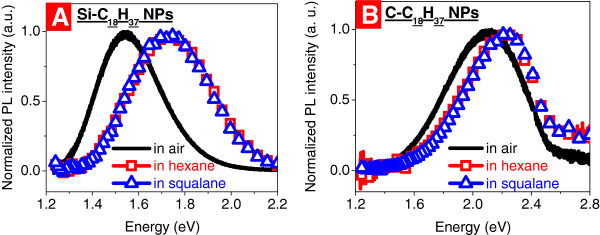
**Room-temperature PL spectra of NPs in air and dispersed in hexane and squalane.** Si-C_18_H_37_ (**A**) and C-C_18_H_37_ (**B**) NPs. Excitation energy of 2.54 eV (**A**) and 3.62 eV (**B**).

Photoluminescence spectra of the Si-C_18_H_37_ and C-C_18_H_37_ NPs in air and in different LPLs (hexane and squalane) are shown in Figure 2A,B, respectively. Significant difference between maximum positions of the PL spectra of both types of NPs in air and in LPLs can be stated. Blue shifts of the PL maximum energy positions of about 190 and 120 meV occur, respectively, for Si-C_18_H_37_ and C-C_18_H_37_ NPs dispersed in the LPLs in comparison with the PL spectra acquired in air. Besides, one can note that the PL spectra of the NPs in hexane and squalane (as well as in decene and octadecene, not shown in Figure 2) are identical. It means that neither chemical composition nor slight difference of dielectric constants of the used LPLs do not influence at all the PL behavior of the NPs. Furthermore, a remarkable broadening of the PL spectra of the Si-C_18_H_37_ and C-C_18_H_37_ NPs after their dispersion in the LPLs takes place, too, at 85 and 60 meV, respectively.

The observed PL changes of the NPs after their dispersion in the LPLs can be explained by reduction of the Förster resonant energy transfer (FRET) efficiency between NPs of different sizes. Indeed, the electronic interaction between the NPs in powder state in air is very strong because they are situated close to each other. It leads to higher FRET probability from small NPs with larger bandgaps toward big NPs having smaller bandgaps. Thus, the small NPs are optically inactive from photo-stimulated emission point of view. In contrast, they are more efficiently separated from each other after their dispersion in LPLs. Indeed, since the probability of the FRET event depends strongly on the distance *D* between two particles (approximately *D*^−6^), the resulting probability of the energy transfer in this case is dramatically reduced. Thus, the probability of the radiative recombination of the photoexcited charge carriers in the smaller NPs is considerably enhanced. Consequently, they become optically active and give their contribution in the PL spectrum resulting in the observed blue shift and broadening. Since no influence of the LPL nature on PL properties of the NPs dispersed in them was observed (see Figure 2 and corresponding discussion above), the FRET mechanism appears as the most preponderant.

**Figure 3:**
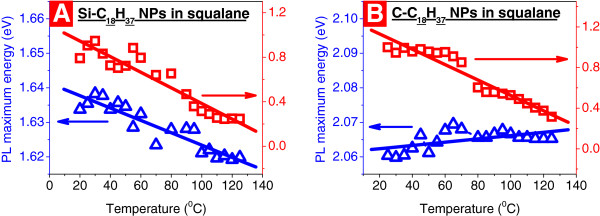
**Temperature dependences of PL maximum energy position (left) and the integral PL intensity (right).** Si-C_18_H_37_ (**A**) and C-C_18_H_37_ (**B**) NPs dispersed in squalane. Excitation energy is 2.54 eV.

Figure 3 shows the influence of ambient temperature on PL properties of the Si-C_18_H_37_ and C-C_18_H_37_ NPs dispersed in squalane (Figure 3A,B, respectively). Heating of the colloidal solutions in 20°C to 100°C temperature range leads to PL quenching for both Si-C_18_H_37_ and C-C_18_H_37_ NPs because the probability of non-radiative recombination of charge carriers due to temperature-induced dissociation of excitons increases. However, the thermal effect on spectral shifts of the PL maxima of the Si-C_18_H_37_ and C-C_18_H_37_ NPs are quite different, which are −0.18 and + 0.05 meV/°C, respectively. As one can see, the Si-based NPs are about three times more sensitive than the C-based NPs. In general, the signs and values of the observed thermal sensitivity are conditioned by more or less important influence of temperature on (1) energy position of the electronic states involved in radiative transitions and (2) their density-dependent population by the photogenerated charge carriers. Obviously, both these reasons seem to play different roles for the Si-C_18_H_37_ and C-C_18_H_37_ NPs.

**Figure 4:**
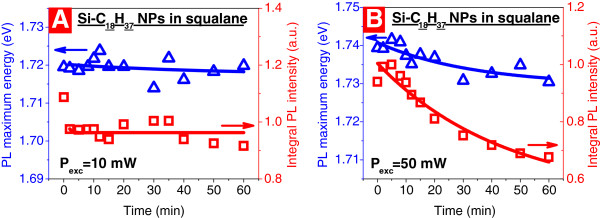
**Dependence of the PL maximum energy position (left) and the integral PL intensity (right).** Si-C_18_H_37_NPs dispersed in squalane upon irradiation time at different excitation power: 10 mW (**A**) and 50 mW (**B**). Room temperature, excitation energy is 2.54 eV.

Figure 4 gives an idea on the photostability of Si-C_18_H_37_ NPs dispersed in squalane at two excitation power levels: 10 mW (Figure 4A) and 50 mW (Figure 4B). At a lower excitation power, continuous laser irradiation for 60 min does not influence both intensity and maximum position of the PL maxima, while quite remarkable photobleaching effect is observed under the higher excitation power. It means that starting from a given threshold of the photoexciting power, squalane with its relatively low thermal conductivity (120 mW/m·K) does not ensure an efficient heat evacuation from the luminescing NPs. Consequently, the laser-induced heating of the NPs takes place, and photochemical reactions between the NPs and squalane are responsible for the observed photobleaching effect.

## Conclusions

Surface chemical modification of Si and C NPs by alkyl chains has succeeded. Perfectly stable and homogeneous colloidal solutions of these NPs in LPLs were obtained. Influence of LPLs, ambient temperature, and irradiation time on PL properties of the NPs was investigated. The linear temperature-dependent integrated PL intensity and spectral position paves the way for the application of this kind of colloidal solutions as temperature-sensitive media. Furthermore, the use of photoexciting power values under a critical threshold (for example, 10 mW for Si-C_18_H_37_ NPs dispersed in squalane) ensures necessary photostability of the colloidal solutions for temperature estimation applications.

## Competing interests

The authors declare that they have no competing interests.

## Authors’ contributions

YVR performed the PL measurements and participated in the discussion of the obtained results. SAA prepared original and chemically modified nanoparticles and colloidal solutions. VVL led the PL data analysis and contributed to the discussion of the obtained results. GB participated in the discussion of the obtained results. J-MB participated in discussion of the obtained results, contributed to the PL data analysis, and coordinated the project. All authors participated in the writing of the submitted and revised versions of the manuscript. All authors read and approved the final manuscript.

## Authors’ information

YVR has obtained his M.S. degree in Solid State Physics in 1997 and his Ph.D. degree in the field of Semiconductor Nanostructures at M.V. Lomonosov Moscow State University in 2007. In 2008, he was invited at Helmholtz-Zentrum Berlin (HZB) in the framework of DAAD fellowship. In 2009, he was employed as a young scientific researcher at the P.N. Lebedev Physical Institute of Russian Academy of Sciences. Since 2012, he has a postdoctoral position at Lyon Institute of Nanotechnologies. His main scientific interest focuses on spectroscopic investigations based on silicon semiconductor nanostructures. VVL has received his M.S. degree in Semiconductor Physics and Electronics from Kiev National Shevchenko University in 1995 and his Ph.D. degree in the field of Integrated Electronic Devices from Ecole Centrale de Lyon (France) in 1998. He was employed as a scientific researcher by the French National Center of Scientific Researches in 2002. Currently, he works at Lyon Institute of Nanotechnologies in the Spectroscopies of Nanomaterials team. His main scientific interest is focused on elaboration, spectroscopic studies of physicochemical properties and multidisciplinary applications of nanomaterials of the IVth group. J-MB obtained his Ph.D. degree in 1996 at the University of Montpellier in the field of Condensed Matter. After two postdoctoral positions in Grenoble, he joined INSA of Lyon as an associate professor in 1999. Currently, he is conducting his research activities in the Lyon Institute of Nanotechnology. His main scientific activity focuses on spectroscopic characterizations of semiconductors nanostructures. SAA received his master's degree in Chemistry from Kiev National Taras Shevchenko University in 1998 and then his Ph.D. degree in Chemistry at the same university in 2003 for his work on the Immobilization of Organic Acids on Silica Gel Surface, Thermochemical and Catalytic Properties of Materials Obtained. Currently, SAA is working as an associate professor in the Chemistry Faculty of the same university. From 2004, SAA has close scientific collaboration with INSA Lyon (France); he participated in European projects such as INTAS, IRSES, and LST. Fields of his research interests are as follows: surface chemistry of nanostructured materials (semiconductors, inorganic oxides), surface functionalization and characterization, application of nanostructures in LDI mass-spectrometry, sensors, catalysis.
